# Trends and Survival Outcomes of Lung Cancer Surgery in South Korea, 2015–2019

**DOI:** 10.1111/1759-7714.70247

**Published:** 2026-01-22

**Authors:** Chanwoo Kim, Kyoung Eun Yeob, Hee‐Sung Kim, Se Eun Park, Jae Yeon Kim, Pankaj Kumar Chaturvedi, Hee Soo Yang, So Young Kim, Jong Hyock Park, Dohun Kim

**Affiliations:** ^1^ College of Medicine Chungbuk National University Cheongju Republic of Korea; ^2^ DK Lab Chungbuk National University Cheongju Republic of Korea; ^3^ Institute of Health and Science Convergence Chungbuk National University Cheongju Republic of Korea; ^4^ Department of Internal Medicine Chungbuk National University College of Medicine Cheongju Republic of Korea; ^5^ Department of Public Health and Preventive Medicine Chungbuk National University Hospital Cheongju Republic of Korea; ^6^ Department of Thoracic and Cardiovascular Surgery Chungbuk National University and Chungbuk National University Hospital Cheongju Republic of Korea

**Keywords:** age groups, lung neoplasms, surgery, survival, trend analysis

## Abstract

**Purpose:**

Lung cancer remains the leading cause of cancer‐related deaths in South Korea, yet a comprehensive evaluation that encompasses evolving patterns of operative choice and their impact on survival outcomes by pathological factors and surgery type is lacking.

**Methods:**

We included 36 663 patients who underwent curative lung cancer resection between 2015 and 2019. Surgical procedures were categorized as pneumonectomy, lobectomy, segmentectomy, or wedge resection, and tumors were staged according to the Surveillance, Epidemiology, and End Results classification scheme. Temporal trends in procedure frequency and age‐group distribution were assessed using trend analyses. Overall survival was estimated by Kaplan–Meier analysis, and independent prognostic factors were identified using multivariable Cox proportional hazards models.

**Results:**

Lobectomy remained the most common operation (78.3%), while the use of segmentectomy and wedge resection increased and that of pneumonectomy declined significantly (all *P* for trend < 0.0001). The proportion of patients aged ≥ 76 years who received surgery rose (trend *p* < 0.0001). Survival was highest following segmentectomy and lobectomy across all age groups and stages. In age group–specific analyses, lobectomy conferred best survival outcomes in the 46–75‐year group (adjusted hazard ratio [aHR], 0.789; 95% confidence interval [CI], 0.734–0.849), whereas segmentectomy yielded favorable survival in the ≥ 76‐year group (aHR, 0.808; 95% CI, 0.676–0.967).

**Conclusion:**

Between 2015 and 2019, the frequency of sublobar resections increased. Segmentectomy conferred the highest survival benefit in patients aged ≥ 76 years, whereas lobectomy was more favorable in patients aged ≤ 75 years, underscoring the importance of tailoring surgical choice to age group.

## Graphical abstract

Among 36 663 Korean patients undergoing curative lung cancer resection (2015–2019), the use of segmentectomy and wedge resection increased while pneumonectomy declined. In multivariable Cox models, lobectomy was associated with the most favorable survival in patients aged 46–75 years (adjusted hazard ratio [aHR] 0.789; 95% CI 0.734–0.849), whereas segmentectomy was most favorable in patients aged ≥ 76 years (aHR 0.808; 95% CI 0.676–0.967), supporting age‐tailored procedure selection.

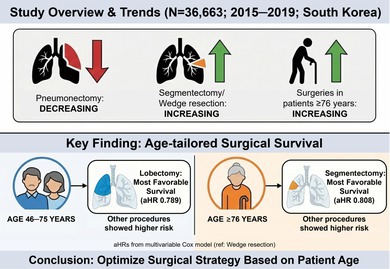

**Abbreviations**
aHR: adjusted hazard ratio
CCI: Charlson Comorbidity Index
CI: confidence interval
CTx: chemotherapy
DM: diabetes mellitus
HR: hazard ratio
HTN: hypertension
ICD‐10: International Classification of Diseases, Tenth Revision
KCCR: Korean Central Cancer Registry
KNHIS: Korean National Health Insurance Service
Ref: reference category
RTx: radiotherapy
SEER: Surveillance, Epidemiology, and End Results

## 1 Introduction

Cancer remains the leading cause of death in South Korea, and among all cancers, lung cancer continues to carry the highest mortality rate despite advances in early detection and treatment [1, 2, 3]. The burden of disease is intensifying with the continued growth of the aging population, particularly among individuals aged ≥ 65 years [4, 5]. Surgical resection is still regarded as the primary curative option for early‐stage non–small‐cell lung cancer. Over the past two decades, the overall rate of lung cancer surgeries in South Korea has shown a steady increase [6]. This upward trend has been accompanied by notable shifts in surgical choices, reflecting changes in the sociodemographic profile of patients undergoing treatment.

Identifying trends in the surgical management of lung cancer—and further, examining their association with survival outcomes—is essential. Previous studies suggest that access to surgery and treatment decisions differ substantially by age, income, geographic region, and the presence of comorbidities [7]. Vulnerable populations, including the elderly, low‐income individuals, and those residing in rural areas, are less likely to receive surgical care [8]. They also face reduced chances of undergoing standard procedures such as lobectomy [8]. Moreover, survival outcomes associated with different types of surgery may vary depending on patient characteristics such as age and other sociodemographic factors [9, 10, 11]. These findings underscore the need for a comprehensive evaluation not only of the volume of surgeries but also the evolving patterns of surgical choice and their influence on clinical outcomes.

To address this gap in the current knowledge, we analyzed lung cancer surgery trends in South Korea from 2015–2019. While many previous Korean studies have relied solely on Korean National Health Insurance Service (KNHIS) data to characterize broad surgical patterns, our study integrates detailed pathology information to evaluate survival outcomes by pathological type and stage. By adopting this integrated approach, we move beyond descriptive trends analysis and seek to identify the specific factors according to pathologic stage that influence survival among lung cancer patients undergoing surgery.

## 2 Methods

### 2.1 Study Setting and Data Source

#### 2.1.1 Korean National Health Insurance Service

For our analysis, we used data from the KNHIS, which provides universal health coverage for nearly the entire Korean population. KNHIS operates on a fee‐for‐service reimbursement system and collects comprehensive information necessary for claims processing; as such, the KNHIS database contains all data required for reimbursement, including diagnostic codes; the specifics of inpatient and outpatient services usage; and a detailed list of diagnostic tests, procedures, other medical treatments, and prescription medications. It also includes data on comorbidities (e.g., hypertension, diabetes mellitus) and mortality, with death information derived from Ministry of Public Administration and Security records and cross‐validated using the date of health insurance qualification termination. Additionally, the KNHIS provides demographic information such as age, sex, residential area, and income level (as indicated by insurance premium tiers). The KNHIS database has been widely used in epidemiological and health policy research [12].

#### 2.1.2 Cancer Registration System in the Republic of Korea

The Korean Central Cancer Registry (KCCR), a government‐sponsored, nationwide cancer registry, was instituted in 1980. This database is one of the most reliable sources of population‐based cancer data in Korea, and it currently covers more than 90% of new cancer cases nationwide [13]. Therefore, the KCCR data have an advantage in that they reflect the entire number of lung cancer cases. The KCCR collects data such as patient sex, age at diagnosis, date of diagnosis, cancer site, surveillance, epidemiology, and summary stage. The Surveillance, Epidemiology, and End Results (SEER) stage of individual cases has been published by the KCCR since 2006. The SEER stage at diagnosis was classified as localized (invasive cancer limited to the organ of origin), regional (tumor extension beyond the limits of the organ of origin), distant (spread to distant areas from the primary tumor), or unknown [14].

### 2.2 Study Populations

We obtained data from January 1, 2015, to December 31, 2019. Patients diagnosed with lung cancer were identified using the International Classification of Diseases, Tenth Revision (ICD‐10), code C34. Among these patients, those who underwent lung cancer surgery between January 1, 2015, and December 31, 2019, were included in the present study. Patients < 20 years of age at the time of surgery were excluded. All subjects were followed until death or for ≥ 5 years after surgery, ending on December 31, 2024, whichever came first. Consequently, a total of 36 663 patients who underwent lung cancer surgery constituted the study population.

### 2.3 Definition of Surgery Type and Pathologic Stage

Lung cancer surgical procedures were identified using the KNHIS reimbursement codes O1400–O1405, O1410, O1421–O1424, and O1431–O1432. Surgical procedures were classified into four types: pneumonectomy, lobectomy, segmentectomy, and wedge resection. Patients who underwent exploratory thoracotomy (KNHIS code O1360) were excluded from the analysis. As mentioned, pathologic stage was categorized based on the SEER summary stage classification: localized (SEER 1: confined to the primary site only), regional (SEER 2: direct extension beyond the primary site only), distant (SEER 7: involvement of distant sites and/or distant lymph nodes), or unknown (SEER 9: summary stage cannot be determined from available information).

We included only patients who underwent curative surgery, and SEER stage 7 cases were included because clinical and pathological staging can differ before and after surgery. In addition, patients with single adrenal or brain metastasis were considered eligible for curative resection despite being classified as SEER 7. Finally, although multiple ground‐glass opacities are recorded as SEER 7, they are often regarded as multiple primary lung cancers, for which surgical resection is commonly performed. Therefore, we considered these cases as having undergone curative surgery.

### 2.4 Variables and Statistical Analysis

Patient characteristics of interest included sociodemographic factors (age, sex, income level), comorbidities (diabetes mellitus, hypertension, other cancers, Charlson Comorbidity Index [CCI]), cancer‐related variables (pathological type and pathologic stage), and treatment details (surgery type, chemotherapy, and radiotherapy). Patients were categorized into three groups according to their age at the time of surgery: 20–45 years (young‐age group), 46–75 years (middle‐age group), and ≥ 76 years (older‐age group). Separately, they were divided into six categories of income (medical aid beneficiaries and five income quintiles [1Q–5Q]) based on their national health insurance premium, reflecting household income. Among the established quintiles, the first quintile (1Q) represents the lowest income group among insured individuals, while the fifth quintile (5Q) represents the highest. Descriptive statistics were used to characterize surgery patterns according to age group. The annual P for trend was determined with a Wilcoxon‐type test for trend across ordered groups. The Kaplan–Meier method with the log‐rank test and Cox proportional hazards regression were used for survival outcomes. All statistical analyses were performed using SAS software version 9.4 (SAS Institute Inc., Cary, NC, USA), and differences were considered statistically significant at two‐sided *p*‐values of ≤ 0.05.

### 2.5 Ethical Statement

The need for informed consent was waived, and the study protocol was approved by the Institutional Review Board of Chungbuk National University (CBNU‐2024‐A‐0048). Our study followed the Strengthening the Reporting of Observational Studies in Epidemiology reporting guideline and was performed in accordance with the principles of the Declaration of Helsinki.

## 3 Results

Among the 36 663 patients included in this study, most (78.3%) belonged to the middle‐age group (46–75 years) (Table 1). Men totaled 60.9% of the study population. The proportion of medical aid beneficiaries was the lowest (4.3%), whereas individuals in the highest income quintile (5Q) accounted for the largest proportion (33.8%) of participants stratified according to income. Hypertension (56.9%) and diabetes mellitus (44.8%) were the most common comorbidities, with prevalence increasing progressively with age across the three age groups. In the middle‐age (46–75 years) and older‐age (≥ 76 years) groups, the proportion of patients with a CCI of ≥ 5 was highest at 44.4% and 56.4%, respectively. In contrast, in the young‐age group (20–45 years), the CCI 0–2 category accounted for the largest proportion, at 40.4%. Adenocarcinoma was the most common histological type (56.4%), followed by squamous cell carcinoma (20.7%) and others (22.9%). Regarding pathological stage, localized (53.6%) and regional (38.0%) stages were most prevalent. Lobectomy was the most frequently performed surgical procedure (78.3%), followed by wedge resection (12.9%). During the follow‐up period, 27.1% of patients received chemotherapy, and 9.0% of patients received radiotherapy.

**TABLE 1:** Basic characteristics of the study population (*n* = 36 663).

Variables	Age groups							
	Overall (*n* = 36 663)		20–45 (*n* = 1046)		46–75 (*n* = 28 710)		≥ 76 (*n* = 6907)	
	*n*	(%)	*n*	(%)	*n*	(%)	*n*	(%)
Sex								
Male	22 310	(60.9)	454	(43.4)	17 255	(60.1)	4601	(66.6)
Female	14 353	(39.1)	592	(56.6)	11 455	(39.9)	2306	(33.4)
Income								
Medical aid	1579	(4.3)	15	(1.4)	1133	(3.9)	431	(6.2)
1Q	5218	(14.2)	134	(12.8)	4306	(15.0)	778	(11.3)
2Q	4299	(11.7)	115	(11.0)	3692	(12.9)	492	(7.1)
3Q	5203	(14.2)	177	(16.9)	4322	(15.1)	704	(10.2)
4Q	7413	(20.2)	239	(22.8)	5901	(20.6)	1273	(18.4)
5Q	12 398	(33.8)	337	(32.2)	8938	(31.1)	3123	(45.2)
Unknown	553	(1.5)	29	(2.8)	418	(1.5)	106	(1.5)
Comorbidities								
DM	16 440	(44.8)	173	(16.5)	12 457	(43.4)	3810	(55.2)
HTN	20 879	(56.9)	114	(10.9)	15 476	(53.9)	5289	(76.6)
Other cancer	6170	(16.8)	99	(9.5)	4566	(15.9)	1505	(21.8)
CCI								
0–2	9514	(25.9)	423	(40.4)	7822	(27.2)	1269	(18.4)
3–4	8462	(23.1)	187	(17.9)	6622	(23.1)	1653	(23.9)
≥ 5	16 906	(46.1)	272	(26.0)	12 739	(44.4)	3895	(56.4)
Unknown	1781	(4.9)	164	(15.7)	1527	(5.3)	90	(1.3)
Pathological type								
Adenocarcinoma	20 692	(56.4)	719	(68.7)	16 541	(57.6)	3432	(49.7)
Squamous cell carcinoma	7590	(20.7)	46	(4.4)	5546	(19.3)	1998	(28.9)
Others	8381	(22.9)	281	(26.9)	6623	(23.1)	1477	(21.4)
Pathologic stage								
Localized	19 665	(53.6)	583	(55.7)	15 395	(53.6)	3687	(53.4)
Regional	13 926	(38.0)	347	(33.2)	10 945	(38.1)	2634	(38.1)
Distant	2039	(5.6)	90	(8.6)	1595	(5.6)	354	(5.1)
Unknown	1033	(2.8)	26	(2.5)	775	(2.7)	232	(3.4)
Surgery type								
Wedge resection	4742	(12.9)	155	(14.8)	3340	(11.6)	1247	(18.1)
Segmentectomy	2544	(6.9)	75	(7.2)	1963	(6.8)	506	(7.3)
Lobectomy	28 700	(78.3)	798	(76.3)	22 807	(79.4)	5095	(73.8)
Pneumonectomy	677	(1.8)	18	(1.7)	600	(2.1)	59	(0.9)
Non‐surgical treatment								
CTx	9950	(27.1)	279	(26.7)	8535	(29.7)	1136	(16.4)
RTx	3316	(9.0)	120	(11.5)	2726	(9.5)	470	(6.8)

Abbreviations: CCI, Charlson Comorbidity Index; CTx, chemotherapy; DM, diabetes mellitus; HTN, hypertension; RTx, radiotherapy.

**FIGURE 1:**
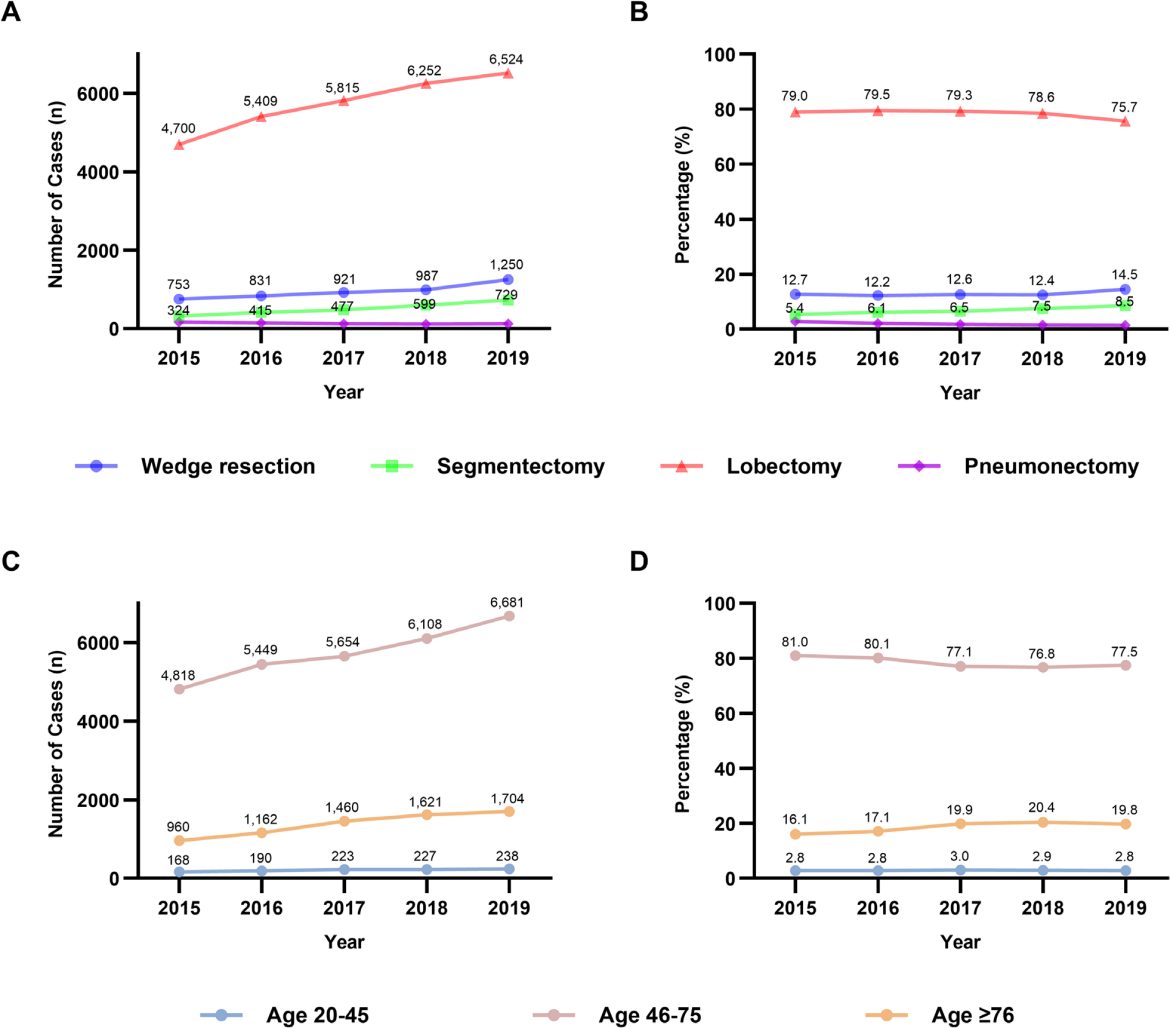
Trends in lung cancer surgery in South Korea. (A) Annual number of lung cancer resections by surgical type. (B) Proportion of lung cancer resections by surgical type. (C) Annual number of lung cancer resections by patient age group. (D) Proportion of lung cancer resections by patient age group.

There was a statistically significant trend in the temporal distribution of surgery type for lung cancer (*P* for trend < 0.0001), with increasing numbers of wedge resection, segmentectomy, and lobectomy cases (all *P* for trend < 0.0001), while pneumonectomy cases decreased over time (*P* for trend < 0.0001) (Figure 1A). Similarly, the proportion of each surgical type showed a significant temporal trend. The proportions of wedge resection and segmentectomy increased (*P* for trend < 0.0001 for both), whereas those of lobectomy and pneumonectomy decreased (*P* for trend < 0.0001 for both) (Figure 1B). Regarding age group distribution over time, the number of cases increased significantly in the middle‐age and older‐age groups (*P* for trend < 0.0001 for both), while there was no significant change in the young‐age group (*P* for trend = 0.8454) (Figure 1C). When considering the proportion of patients undergoing surgery in each age group, the middle‐age group showed a decreasing trend (*P* for trend < 0.0001), the older‐age group showed an increasing trend (*P* for trend < 0.0001), and the young‐age group remained unchanged (*P* for trend = 0.8454) (Figure 1D).

**FIGURE 2:**
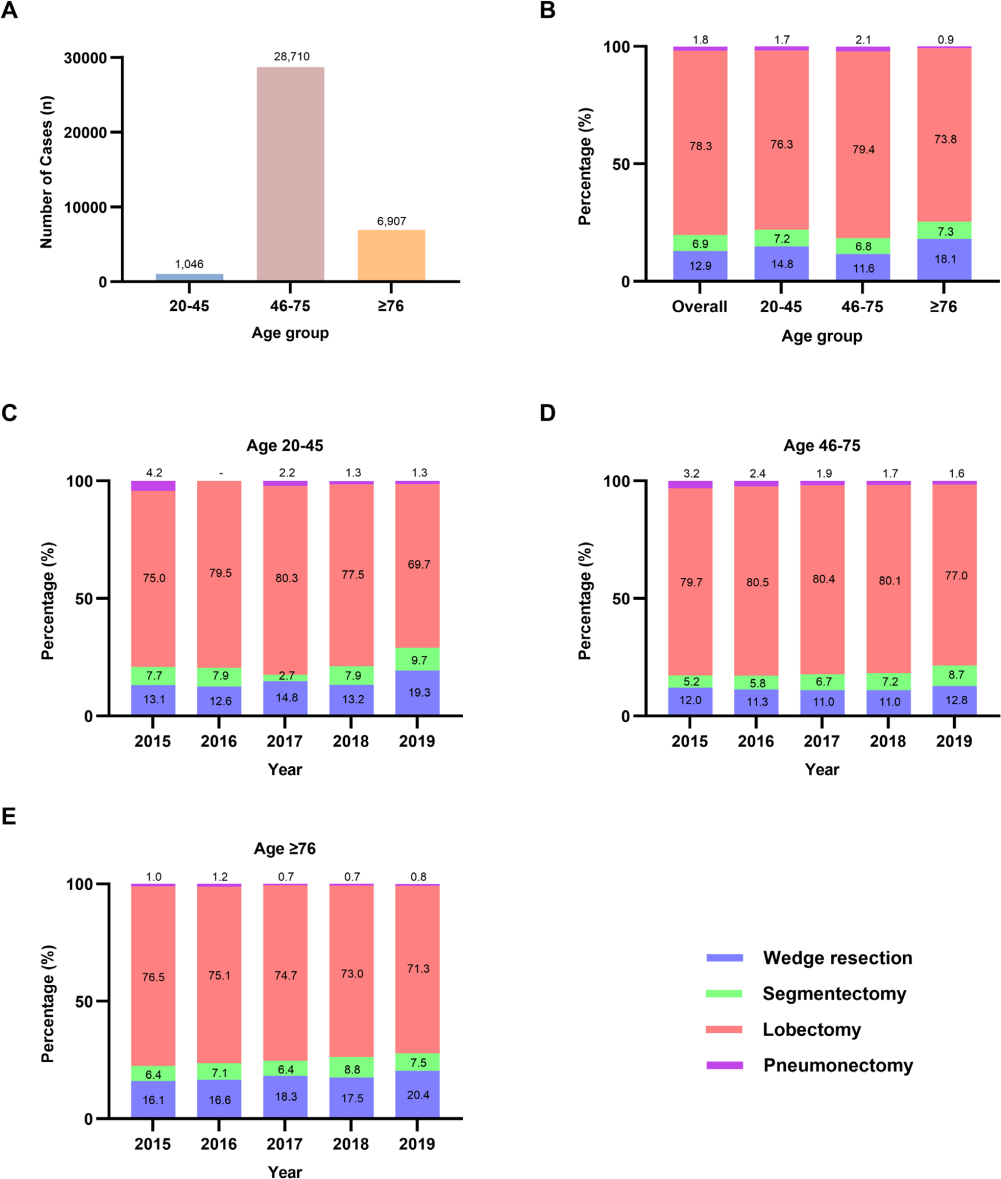
Distribution of curative lung cancer resections by age group. (A) Total number of resections by age group. (B) Overall proportion of resection types by age group. (C) Annual proportion of resection types in patients aged 20–45 years. (D) Annual proportion of resection types in patients aged 46–75 years. (E) Annual proportion of resection types in patients aged ≥ 76 years.

The distribution of curative resections by age group is illustrated in Figure 2A–E. The number of surgeries was highest in the middle‐age group (*n* = 28 710), followed by the older‐age (*n* = 6907) and young‐age (*n* = 1046) groups (Figure 2A). Across all age groups, lobectomy was the most commonly performed surgical procedure, followed by wedge resection, segmentectomy, and pneumonectomy (Figure 2B). In the young‐age group, there was no statistically significant temporal trend in the distribution of surgery types (*P* for trend = 0.1229) (Figure 2C). In the middle‐age group, wedge resection (*P* for trend = 0.0055) and segmentectomy (*P* for trend < 0.0001) showed increasing trends, while the trends for lobectomy (*P* for trend = 0.0001) and pneumonectomy (*P* for trend < 0.0001) decreased over time (Figure 2D). In the older‐age group, wedge resection increased (*P* for trend = 0.0005) and lobectomy decreased (*P* for trend = 0.0010), whereas there were no significant changes in the proportions of segmentectomy (*P* for trend = 0.3182) and pneumonectomy (*P* for trend = 0.3003) (Figure 2E).

**FIGURE 3:**
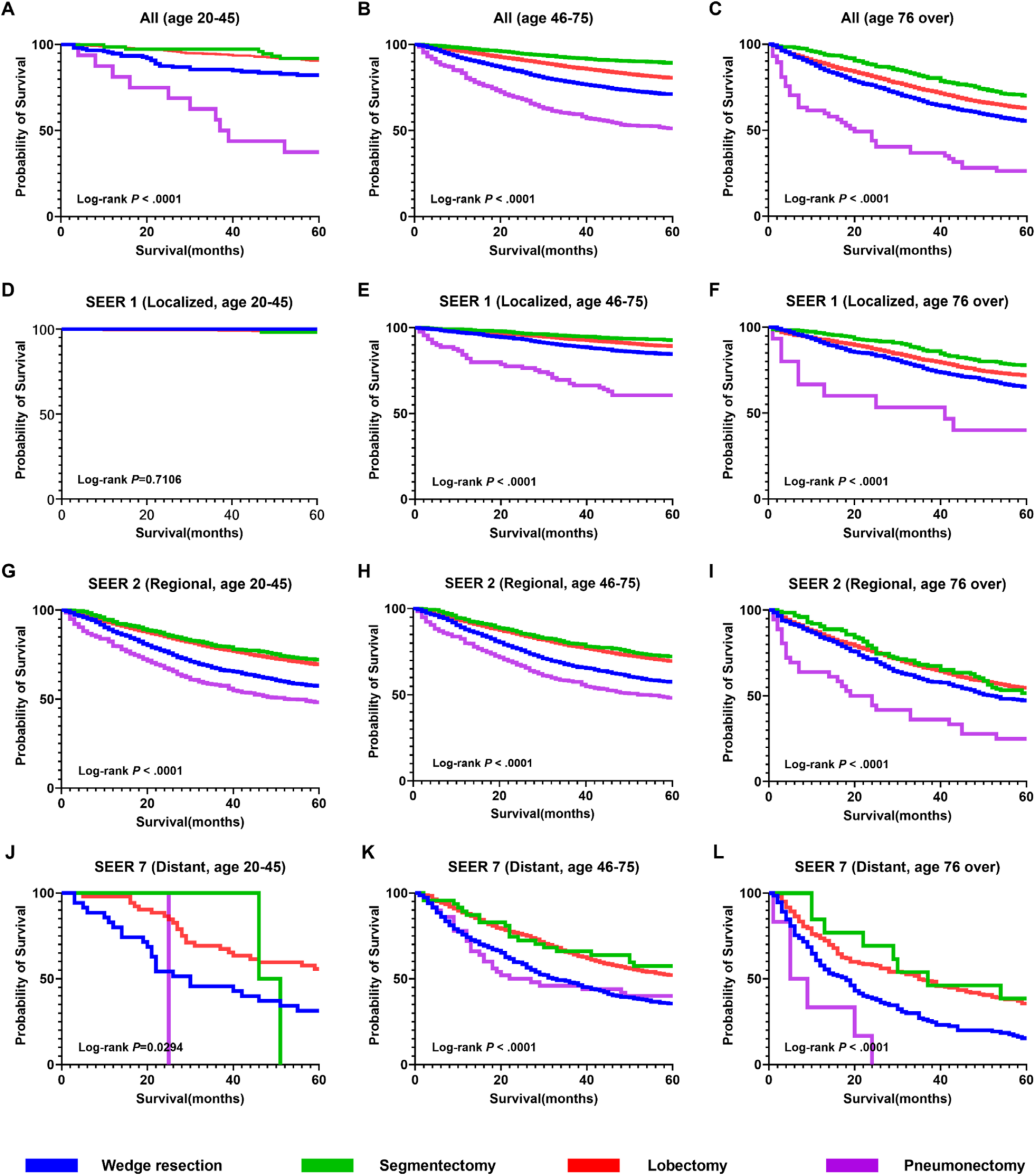
Kaplan–Meier survival curves for lung cancer surgery patients by age group and SEER stage. (A) All patients aged 20–45 years (*n* = 1020). (B) All patients aged 46–75 years (*n* = 27 935). (C) All patients aged ≥ 76 years (*n* = 6675). (D) Localized disease (SEER 1) in patients aged 20–45 years (*n* = 583). (E) Localized disease (SEER 1) in patients aged 46–75 years (*n* = 15 395). (F) Localized disease (SEER 1) in patients aged ≥ 76 years (*n* = 3687). (G) Regional disease (SEER 2) in patients aged 20–45 years (*n* = 347). (H) Regional disease (SEER 2) in patients aged 46–75 years (*n* = 10 945). (I) Regional disease (SEER 2) in patients aged ≥ 76 years (*n* = 2634). (J) Distant disease (SEER 7) in patients aged 20–45 years (*n* = 90). (K) Distant disease (SEER 7) in patients aged 46–75 years (*n* = 1595). (L) Distant disease (SEER 7) in patients aged ≥ 76 years (*n* = 354). SEER, Surveillance, Epidemiology, and End Results.

Survival analysis according to age group and pathological stage demonstrated significant differences in overall survival across surgery types (Figure 3A–C). Segmentectomy and lobectomy were associated with the most favorable survival outcomes, followed by wedge resection, while pneumonectomy led to the poorest outcomes in all age groups. In the young‐age group with localized disease, 5‐year survival rates were close to 100% across all surgical types, and the difference was not statistically significant (log‐rank *p* = 0.7106) (Figure 3D). In both the middle‐ and older‐age groups with localized disease, segmentectomy was associated with the best survival outcomes (log‐rank *p* < 0.0001 for both) (Figure 3E,F). In patients with regional and distant disease across all age groups, those undergoing segmentectomy or lobectomy demonstrated better survival than those undergoing wedge resection or pneumonectomy (Figure 3G–L).

Cox proportional hazards regression analysis was performed to identify prognostic factors stratified by age group (Table 2). Male sex was significantly associated with worse survival in the overall cohort (adjusted hazard ratio [aHR], 1.335; 95% confidence interval [CI], 1.263–1.412). Low‐income groups, including medical aid and lower income quintiles (4Q), generally showed poorer survival compared to the highest income group (5Q), with statistical significance observed in the overall cohort (aHR, 1.198; 95% CI, 1.095–1.311 and aHR, 1.074; 95% CI, 1.011–1.140), respectively. Among comorbidities, diabetes mellitus was associated with a significantly increased risk of mortality in the overall cohort (aHR, 1.070; 95% CI, 1.021–1.121). For CCI, survival was not significantly different in patients with a score of 3–4 compared to those with 0–2, but patients with a score of ≥ 5 showed significantly worse survival (aHR, 1.200; 95% CI, 1.126–1.279). Histologically, patients with squamous cell carcinoma (aHR, 1.180; 95% CI, 1.120–1.243) and other pathological types (aHR, 1.351; 95% CI, 1.278–1.427) had poorer survival compared to those with adenocarcinoma. Regional (aHR, 1.318; 95% CI, 1.255–1.384) and distant stages (aHR, 1.692; 95% CI, 1.575–1.818) were also associated with significantly higher mortality than localized disease. Regarding surgery type, segmentectomy (aHR, 0.822; 95% CI, 0.733–0.921) and lobectomy (aHR, 0.828; 95% CI, 0.781–0.877) were both associated with favorable survival outcomes, whereas pneumonectomy carried the highest risk of mortality (aHR, 1.225; 95% CI, 1.085–1.383).

**TABLE 2:** Cox regression analysis to explore prognostic factors by age group.

Variables	a. Overall (*n* = 36 663)				b. 20–45 (*n* = 1046)				c. 46–75 (*n* = 28 710)				d. ≥ 76 (*n* = 6907)			
	Univariate		Multivariate		Univariate		Multivariate		Univariate		Multivariate		Univariate		Multivariate	
	HR (95% CI)	*p*	aHR (95% CI)	*p*	HR (95% CI)	*p*	aHR (95% CI)	*p*	HR (95% CI)	*p*	aHR (95% CI)	*p*	HR (95% CI)	*p*	aHR (95% CI)	*p*
Sex		< 0.0001		< 0.0001		0.0378		0.0514		< 0.0001		< 0.0001		< 0.0001		< 0.0001
Male	1.391 (1.32–1.466)		1.335 (1.263–1.412)		1.468 (1.022–2.108)		1.502 (0.997–2.261)		1.384 (1.297–1.476)		1.321 (1.234–1.415)		1.402 (1.277–1.539)		1.367 (1.236–1.512)	
Female	Ref		Ref		Ref		Ref		Ref		Ref		Ref		Ref	
Income		0.0037		0.0007		0.2546		0.2142		0.0021		0.0042		0.2629		0.1629
Medical Aid	1.163 (1.064–1.271)		1.198 (1.095–1.311)		0.278 (0.038–2.043)		0.307 (0.040–2.373)		1.253 (1.122–1.399)		1.248 (1.116–1.395)		1.048 (0.900–1.222)		1.127 (0.966–1.315)	
1Q	1.041 (0.973–1.115)		1.037 (0.969–1.111)		0.953 (0.511–1.778)		0.669 (0.328–1.365)		1.049 (0.966–1.140)		1.013 (0.932–1.101)		1.110 (0.979–1.258)		1.107 (0.976–1.256)	
2Q	1.065 (0.991–1.144)		1.055 (0.981–1.135)		1.101 (0.581–2.085)		0.962 (0.447–2.073)		1.110 (1.020–1.208)		1.052 (0.966–1.145)		1.035 (0.886–1.209)		1.039 (0.888–1.215)	
3Q	1.011 (0.945–1.082)		0.993 (0.927–1.063)		0.825 (0.480–1.417)		0.795 (0.449–1.406)		1.064 (0.980–1.154)		1.009 (0.929–1.095)		0.966 (0.848–1.100)		0.955 (0.838–1.088)	
4Q	1.088 (1.025–1.155)		1.074 (1.011–1.140)		1.502 (0.910–2.479)		1.523 (0.844–2.749)		1.096 (1.017–1.181)		1.051 (0.975–1.133)		1.112 (1.003–1.233)		1.103 (0.995–1.223)	
5Q	Ref		Ref		Ref		Ref		Ref		Ref		Ref		Ref	
Comorbidities																
DM (Ref = No disease)	1.153 (1.105–1.203)	< 0.0001	1.070 (1.021–1.121)	0.0047	1.058 (0.702–1.593)	0.7883	0.653 (0.400–1.066)	0.0881	1.173 (1.114–1.235)	< 0.0001	1.088 (1.028–1.151)	0.0035	1.089 (1.008–1.177)	0.0310	1.039 (0.955–1.13)	0.3787
HTN (Ref = No disease)	1.034 (0.989–1.080)	0.572	0.986 (0.941–1.033)	0.5592	1.225 (0.732–2.049)	0.4394	1.048 (0.546–2.013)	0.8881	1.015 (0.963–1.070)	0.5720	0.966 (0.914–1.020)	0.2152	1.013 (0.927–1.108)	0.7734	1.017 (0.928–1.115)	0.7169
Other cancer (Ref = No disease)	1.042 (0.991–1.096)	0.6433	1.015 (0.964–1.069)	0.5667	1.496 (0.903–2.479)	0.1180	1.319 (0.732–2.376)	0.3564	1.015 (0.953–1.080)	0.6433	0.993 (0.931–1.059)	0.8314	1.070 (0.981–1.167)	0.1284	1.031 (0.942–1.128)	0.5124
CCI		< 0.0001		< 0.0001		0.0530		0.2536		< 0.0001		< 0.0001		< 0.0001		0.0003
0–2	Ref		Ref		Ref		Ref		Ref		Ref		Ref		Ref	
3–4	1.073 (1.000–1.152)		1.042 (0.969–1.120)		1.639 (0.893–3.008)		1.458 (0.725–2.930)		1.081 (0.992–1.177)		1.057 (0.969–1.153)		1.015 (0.890–1.158)		0.995 (0.870–1.138)	
≥ 5	1.286 (1.213–1.364)		1.200 (1.126–1.279)		1.761 (1.114–2.785)		1.519 (0.874–2.640)		1.28 (1.193–1.373)		1.190 (1.104–1.284)		1.252 (1.120–1.399)		1.206 (1.070–1.360)	
Pathological type		< 0.0001		< 0.0001		0.0004		0.0006		< 0.0001		< 0.0001		< 0.0001		< 0.0001
Adenocarcinoma	Ref		Ref		Ref		Ref		Ref		Ref		Ref		Ref	
Squamous cell carcinoma	1.313 (1.251–1.378)		1.180 (1.120–1.243)		3.210 (1.793–5.745)		3.746 (1.844–7.611)		1.327 (1.251–1.408)		1.213 (1.137–1.293)		1.244 (1.142–1.355)		1.110 (1.013–1.217)	
Others	1.419 (1.344–1.498)		1.351 (1.278–1.427)		1.355 (0.903–2.034)		1.567 (0.966–2.540)		1.438 (1.348–1.535)		1.368 (1.281–1.462)		1.384 (1.250–1.533)		1.315 (1.186–1.459)	
Pathologic stage		< 0.0001		< 0.0001		0.1419		0.1568		< 0.0001		< 0.0001		< 0.0001		< 0.0001
Localized	Ref		Ref		Ref		Ref		Ref		Ref		Ref		Ref	
Regional	1.267 (1.209–1.329)		1.318 (1.255–1.384)		2.381 (0.956–5.930)		3.223 (1.180–8.806)		1.313 (1.238–1.392)		1.353 (1.273–1.437)		1.221 (1.125–1.326)		1.234 (1.135–1.342)	
Distant	1.617 (1.510–1.731)		1.692 (1.575–1.818)		2.872 (1.143–7.218)		2.862 (1.050–7.804)		1.635 (1.506–1.775)		1.645 (1.509–1.792)		1.853 (1.616–2.125)		1.870 (1.628–2.147)	
Unknown	1.207 (1.072–1.358)		1.178 (1.046–1.327)		3.199 (0.763–13.421)		2.705 (0.569–12.872)		1.120 (0.963–1.304)		1.091 (0.937–1.270)		1.377 (1.138–1.667)		1.317 (1.087–1.596)	
Surgery type		< 0.0001		< 0.0001		0.0112		0.0379		< 0.0001		< 0.0001		< 0.0001		< 0.0001
Wedge resection	Ref		Ref		Ref		Ref		Ref		Ref		Ref		Ref	
Segmentectomy	0.777 (0.694–0.870)		0.822 (0.733–0.921)		0.615 (0.254–1.491)		0.615 (0.239–1.582)		0.796 (0.685–0.924)		0.847 (0.728–0.985)		0.764 (0.640–0.913)		0.812 (0.679–0.971)	
Lobectomy	0.801 (0.758–0.847)		0.828 (0.781–0.877)		0.552 (0.357–0.855)		0.490 (0.285–0.841)		0.775 (0.723–0.830)		0.792 (0.736–0.852)		0.882 (0.803–0.968)		0.899 (0.817–0.990)	
Pneumonectomy	1.323 (1.177–1.487)		1.225 (1.085–1.383)		1.278 (0.618–2.641)		0.810 (0.315–2.083)		1.247 (1.093–1.422)		1.099 (0.959–1.261)		2.226 (1.637–3.027)		1.958 (1.433–2.675)	

Abbreviations: aHR, adjusted hazard ratio; CCI, Charlson Comorbidity Index; CI, confidence interval; DM, diabetes mellitus; HR, hazard ratio; HTN, hypertension; Ref, reference category.

We also examined detailed age group–specific patterns (Table 2). In terms of income, the medical aid group showed significantly worse survival compared to the highest income quintile (5Q) exclusively in the middle‐age group (aHR, 1.248; 95% CI, 1.116–1.395), while no significant differences were observed in the young‐ or older‐age groups after multivariable adjustment. Regarding comorbidities, diabetes mellitus was associated with significantly higher mortality in the middle‐age group (aHR, 1.088; 95% CI, 1.028–1.151). Similarly, a CCI score of ≥ 5 was significantly associated with poorer survival compared to a score of 0–2 in both the middle‐age (aHR, 1.190; 95% CI, 1.104–1.284) and older‐age (aHR, 1.206; 95% CI, 1.070–1.360) groups. With respect to surgery type, in the middle‐age group, lobectomy (aHR, 0.792; 95% CI, 0.736–0.852) led to more favorable outcomes than segmentectomy (aHR, 0.847; 95% CI, 0.728–0.985). In contrast, in the older‐age group, segmentectomy (aHR, 0.812; 95% CI, 0.679–0.971) conferred a greater survival benefit than lobectomy (aHR, 0.899; 95% CI, 0.817–0.990), suggesting that parenchyma‐saving resection may be a more suitable option in elderly patients.

## 4 Discussion

In our nationwide cohort of 36 663 lung cancer surgery patients treated between 2015 and 2019, 78.3% were in the middle‐age group, 60.9% were male, and 33.8% were in the highest income quintile (5Q). Lobectomy accounted for 78.3% of all procedures, followed by wedge resection (12.9%). Over the study period, the conduct of wedge resection and segmentectomy alike increased markedly (both *p* < 0.0001), while that of pneumonectomy declined (*p* < 0.0001). Survival analyses showed that male sex and low‐income status were both significantly associated with poorer overall survival. Furthermore, segmentectomy and lobectomy yielded the highest overall survival across age groups, with segmentectomy conferring the greatest advantage in localized disease and among patients aged ≥ 76 years.

Despite an overall increase in lung cancer incidence, our analysis demonstrates a clear decline in pneumonectomy use between 2015 and 2019 [1, 2]. This shift reflects both the increasing adoption of sublobar resections, primarily indicated for early‐stage tumors detected through widespread low‐dose computed tomography screening [15, 16], and the growing recognition of the substantial perioperative morbidity and long‐term functional impairment associated with removal of an entire lung [17]. Pneumonectomy carries significantly higher rates of cardiopulmonary complications and perioperative mortality compared to parenchyma‐sparing resections [18, 19], and survivors frequently endure chronic dyspnea, reduced exercise tolerance, and mediastinal shift syndromes—so‐called “post‐pneumonectomy syndrome”—which some authors have described as effectively constituting a secondary disease process in these patients [20, 21]. In contrast, lobectomy and segmentectomy offer comparable oncologic efficacy in early‐stage disease while preserving greater pulmonary reserves [9, 22], making them particularly attractive for elderly or comorbid patients. The declining preference for pneumonectomy thus underscores an evolving paradigm in lung cancer surgery that prioritizes not only survival but also postoperative quality of life by minimizing physiological disruption through parenchyma‐sparing resections.

Meanwhile, the comparative evaluation of lobectomy versus segmentectomy has persisted for decades without reaching a definitive consensus [9, 22]. Lobectomy offers the advantage of lower postoperative recurrence rates [23], whereas segmentectomy preserves pulmonary function to a greater extent [24]. While the JCOG0802/WJOG4607L and CALGB 140503 trials demonstrated the oncologic safety of segmentectomy compared with lobectomy in carefully selected T1aN0 peripheral tumors, our nationwide analysis broadens this context by examining survival patterns across all age groups and SEER stages in real‐world clinical practice [22]. In our temporal analysis of surgical patterns, we observed a gradual increase in the proportion of segmentectomies alongside a corresponding decline in lobectomies. Survival analysis further demonstrated that both lobectomy and segmentectomy conferred superior overall survival compared to wedge resection or pneumonectomy, with segmentectomy associated with a slightly more favorable aHR. When stratified by age group, lobectomy was associated with a more favorable aHR in the young‐ and middle‐age groups, whereas segmentectomy yielded a superior aHR among older patients. These findings suggest that, for young‐ and middle‐aged individuals, prioritizing lobectomy to reduce recurrence may optimize the chance of cure [9], whereas, among elderly patients with reduced baseline pulmonary reserve, prioritizing segmentectomy to conserve lung function may translate into a survival benefit [25]. While current guidelines primarily rely on tumor characteristics, nodal characteristics, and cardiopulmonary function, our results indicate that an age threshold around 75 years may serve as an efficient criterion when selecting between lobectomy and segmentectomy.

In addition, we identified several noteworthy patient‐level characteristics. First, there was a clear aging trend among surgical candidates: the proportion of middle‐aged patients declined over time, whereas the share of older patients steadily increased. This shift likely reflects both overall populations aging and the expansion of early lung cancer screening programs [26]. Second, we observed disparities in incidence and survival by socioeconomic status. Although individuals in the highest income quintile (5Q) made up the largest share of surgical cases, survival was lowest among medical aid beneficiaries. This pattern may reflect higher screening uptake among wealthier groups, which can lead to an apparent overestimation of incidence [27]. Lower‐income patients, by contrast, may experience treatment inaccessibility or delays that worsen their outcomes [6, 8]. Finally, a significant sex‐based survival difference emerged, which may be attributable to the greater prevalence of smoking‐related squamous cell carcinoma among men. In our cohort, squamous cell carcinoma conferred significantly worse survival compared to adenocarcinoma (aHR 1.188; 95% CI, 1.127–1.251), consistent with large‐scale epidemiological findings [28].

This study has several limitations that should be acknowledged. First, although the dataset spans from 2015 to 2019, only patients with at least 5 years of follow‐up were included to ensure reliable survival estimates. Consequently, more recent data were not analyzed. If cases from 2020 onward were incorporated, they would likely reflect the increasing adoption of minimally invasive and robotic techniques and might demonstrate different surgical trends and survival patterns. Second, distinctions between open thoracotomy and video‐assisted thoracoscopic surgery could not be made, as the KNHIS database does not differentiate between these approaches. Moreover, robotic‐assisted thoracic surgery is not reimbursed in South Korea to date and therefore is not captured in claims data, which limited our ability to compare mortality and long‐term survival across different surgical approaches. Third, because this study relied on insurance claims rather than clinical records, important variables such as lung function, inflammatory markers, frailty or activity status, and residual tumor status were unavailable. Therefore, caution is warranted to avoid overinterpreting these findings based on factors not included in this study. Fourth, although we evaluated survival by pathological type and SEER stage, we were unable to determine which surgical procedure conferred the highest survival for a given pathological status. While segmentectomy appeared more favorable than lobectomy in certain subgroups, a direct head‐to‐head comparison was beyond the scope of this study, which aimed to provide a population‐level overview of surgical trends and overall survival outcomes. We hope that future research will further explore this clinically important question.

In conclusion, the observed trends indicate that lung cancer surgeries aside from pneumonectomy are on the rise and the proportion of elderly patients among those undergoing surgery is increasing. Moreover, when assessing survival outcomes according to age group and stage, it was found that, particularly in early‐stage disease and among elderly patients, segmentectomy produced superior survival outcomes. However, lobectomy demonstrated a slightly more favorable effect in middle‐age patients, with this advantage being even more pronounced in young‐age patients. Further randomized controlled trials comparing lobectomy and segmentectomy across different age groups are warranted.

## Author Contributions

All authors had full access to the data in the study and take responsibility for the integrity of the data and the accuracy of the data analysis. Conceptualization: Chanwoo Kim, Kyoung Eun Yeob, Hee‐Sung Kim, Jong Hyock Park, and Dohun Kim. Data curation: Hee‐Sung Kim, So Young Kim, and Jong Hyock Park. Formal analysis: Chanwoo Kim, Kyoung Eun Yeob, and Hee Soo Yang. Project administration: Pankaj Kumar Chaturvedi, Jae Yeon Kim, and Se Eun Park. Visualization: Chanwoo Kim and Kyoung Eun Yeob. Writing – original draft: Chanwoo Kim and Kyoung Eun Yeob. Writing – review and editing: Chanwoo Kim, Pankaj Kumar Chaturvedi, Jae Yeon Kim, Se Eun Park, and Dohun Kim. Supervision: Jong Hyock Park and Dohun Kim.

## Funding

The authors have nothing to report.

## Conflicts of Interest

The authors declare no conflicts of interest.

## Data Availability

The National Health Insurance claim data are not publicly available due to their sensitive nature and are only accessible to qualified researchers upon request to the National Health Insurance Service.
